# Investigation of the Process Conditions for Hydrogen Production by Steam Reforming of Glycerol over Ni/Al_2_O_3_ Catalyst Using Response Surface Methodology (RSM)

**DOI:** 10.3390/ma7032257

**Published:** 2014-03-19

**Authors:** Ali Ebshish, Zahira Yaakob, Yun Hin Taufiq-Yap, Ahmed Bshish

**Affiliations:** 1 Department of Chemical and Process Engineering, Faculty of Engineering, Universiti Kebangsaan Malaysia (UKM), Bangi, Selangor 43600, Malaysia; E-Mail: ahmedbshish@gmail.com; 2 Fuel Cell Institute, Universiti Kebangsaan Malaysia (UKM), Bangi, Selangor 43600, Malaysia; 3 Catalysis Science and Technology Research Centre, Faculty of Science, Universiti Putra Malaysia, UPM Serdang, Selangor 43400, Malaysia; E-Mail: yap@science.upm.edu.my

**Keywords:** glycerol reforming, hydrogen production, response surface methodology, nickel catalyst

## Abstract

In this work; a response surface methodology (RSM) was implemented to investigate the process variables in a hydrogen production system. The effects of five independent variables; namely the temperature (X_1_); the flow rate (X_2_); the catalyst weight (X_3_); the catalyst loading (X_4_) and the glycerol-water molar ratio (X_5_) on the H_2_ yield (Y_1_) and the conversion of glycerol to gaseous products (Y_2_) were explored. Using multiple regression analysis; the experimental results of the H_2_ yield and the glycerol conversion to gases were fit to quadratic polynomial models. The proposed mathematical models have correlated the dependent factors well within the limits that were being examined. The best values of the process variables were a temperature of approximately 600 °C; a feed flow rate of 0.05 mL/min; a catalyst weight of 0.2 g; a catalyst loading of 20% and a glycerol-water molar ratio of approximately 12; where the H_2_ yield was predicted to be 57.6% and the conversion of glycerol was predicted to be 75%. To validate the proposed models; statistical analysis using a two-sample *t*-test was performed; and the results showed that the models could predict the responses satisfactorily within the limits of the variables that were studied.

## 1. Introduction

There has been a greatly increased consumption of energy over the past few decades, because of the growing world population and industrialization [1]. Currently, the main resources that are used for energy production worldwide are fossil fuels, which are being depleted rapidly. Furthermore, the combustion of fossil fuels causes the production of a large amount of greenhouse gases, such as CO_2_ and CH_4_, and toxic gases, such as NO*_x_* and SO_2_. These gases adversely impact the global warming issue and cause acid rain [2]. To solve these problems, intensified efforts have been made to explore a clean, renewable and sustainable energy supply source. Hydrogen can be produced from biomass and is one possible type of renewable energy [3–6]. Furthermore, hydrogen can deliver and store energy in a utilizable form. Furthermore, hydrogen possesses a high energy capacity that can be efficiently utilized in fuel cell applications with no effects on the surroundings. At present, the majority of the hydrogen supply in the market (approximately 98%) is produced from fossil fuels [7]. The production of biodiesel has widely increased, because of a large increase in crude oil prices and because it is an environmentally friendly fuel, resulting in an excess of glycerol being produced as a byproduct from the biodiesel production processes [8,9]. The production of glycerol has tripled in Europe over the past decade and has reached more than 350,000 tons per annum in the United States [10]. One problem concerning the surplus amount of glycerol produced is its utilization in economic ways. Therefore, the utilization of glycerol for the production of hydrogen rather than its disposal will be of great significance in both the biodiesel production economy and the clean renewable energy supply.

Currently, it has been reported that biomass-derived glycerol can be potentially used as a renewable substrate for hydrogen production via the steam reforming process [8,11–16]. The overall steam reforming reaction is shown below [17]:

$$\begin{array}{cc} C_{3}H_{8}O_{3}+3H_{2}O\to 3{\mathrm{CO}}_{2}+{7H}_{2}O & \Delta H_{298}^{0}=+123\text{kJ}/\text{mol} \end{array}$$ (1)

To investigate the effect of the reaction variables on the production of hydrogen, several studies have been performed under a wide range of operating conditions [18–22]. The determination of effective reaction conditions will provide an opportunity for utilizing glycerol to produce hydrogen efficiently. However, in a multivariable process, the traditional approach of changing one factor at a time while the others are kept unchanged for a specific set of experiments is both time consuming and wastes raw materials. Moreover, as the effect of the interaction between variables is not considered in this technique, the true efficient values of the process variables will not be achieved [23].

In response to the above issue, a collection of mathematical and statistical methods should be used to design experiments, to analyze the effect of the separate and interaction variables and to build models that are capable of describing the responses. Response surface methodology (RSM) is an optimization technique that is commonly applied to optimize process variables in various applications because of its simplicity, sensibly high efficiency and comprehensive theory [1,23–27]. In addition, the experimental trials required to evaluate the process variables can be reduced when RSM is implemented [28].

In this study, the investigation of process variables, namely the temperature, the feed flow rate, the catalyst weight, the catalyst loading and the glycerol-water molar ratio, in the steam reforming of glycerol for producing hydrogen has been performed via RSM using a central composite design (CCD). The best numerical values of the reforming process variables were statistically predicted. To the best of our knowledge, there is no published work on exploring the process conditions for glycerol steam reforming to produce hydrogen over a xerogel Ni/Al_2_O_3_ catalyst, which was prepared by a sol-gel process using polyethylene glycol as a surfactant material.

## 2. Experimental Work

### 2.1. Catalyst Preparation

The sol-gel method was implemented to synthesize the Ni/Al_2_O_3_ catalyst [29]. The procedure to prepare the nickel alumina catalyst was as follows: first, 20 g of aluminum isopropoxide (C_9_H_2_AlO_3_) was dissolved in 240 mL of ethanol (C_2_H_5_OH). To obtain a homogeneous solution, the aluminum isopropoxide and ethanol were stirred at 60 °C for 2 h. While stirring the first solution, a second solution containing 2.75 g of nickel nitrate hexahydrate (Ni(NO_3_)_2_·6H_2_O) in 50 mL of ethanol was prepared. Under continuous stirring, approximately 23 g of polyethylene glycol (PEG) were added to the second solution to keep the final mass ratio of nickel nitrate/polyethylene glycol at 0.11. The nickel nitrate and polyethylene glycol solution was then added drop-wise to the first solution. The final solution was stirred at 65 °C for 5 h. After stirring, a solution of water and ethanol at a mass ratio of 1:2 was added to the final solution. A gel was produced upon addition of the ethanol-water solution. The gel was dried in an oven at 110 °C for one week. Finally, the nickel alumina catalyst was calcined at 700 °C for 5 hours.

### 2.2. Catalyst Performance Test

All experiments were conducted over the xerogel Ni/Al_2_O_3_ catalyst to investigate the optimal operating conditions for hydrogen production by the steam reforming of glycerol in a fixed-bed stainless steel reactor (length: 39 mm; inside diameter: 6.35 mm; wall thickness: 0.9 mm). In these experiments, glycerol and water mixed at specific molar ratios were fed to the core of the reactor, where the nickel catalyst was placed over a glass wool support. A programmable syringe pump (PHD 4400, Harvard Apparatus, Holliston, MA, United States) was used to inject the feed solution into the reactor. The feed was vaporized using a pre-heater placed before the reactor. Nitrogen was used as a carrier gas (30 mL/min). The reaction variables were the reaction temperature, the feed flow rate, the catalyst weight, the catalyst loading and the glycerol-water molar ratio, which were verified according to the design of experiments as shown below. Table 1 shows the five mentioned independent variables and their coded values that were employed in the central composite design of the experiments. In all experiments, the catalyst was first pretreated at an elevated temperature of 120 °C for 1 h to remove impurities and was then reduced under hydrogen flow for 2 h. The output stream was cooled down using crushed ice to separate the gas and the liquid products. For optimization purposes, the gas sample of all experiments was analyzed at the first hour of the reaction. The separated gases were sent to an online gas chromatography GC instrument (Clarus 500 Perkin Elmer with FID and TCD detectors, SRI 8610 C, USA) for gas analysis. These experiments were performed using a steam reformer unit available at Fuel Cell Institute, Universiti Kebangsaan, Malaysia.

The responses that were investigated in this work were measured in terms of hydrogen yield and the conversion of glycerol into gaseous products. The response parameters were calculated according to the following equations [17]:

$$\text{Hydrogen Yield},\;\%\;=\frac{\text{Hydrogen moles produced}}{\text{Maximum moles of hydrogen }(=7\times \text{moles of glycerol fed})}\times 100$$ (2)

$$\text{Glycerol conversion to gases,}\%=\frac{\text{C atoms in the gas product stream}}{\text{Total C atoms in the feedstock}}\times 100$$ (3)

## 3. Statistical Design of Experiment

### 3.1. Experimental Design

A five-factor CCD was implemented to investigate the effects of the independent operating variable conditions [X_1_ (temperature: 400–600 °C), X_2_ (feed flow rate: 0.05–0.1 mL/min), X_3_ (catalyst weight: 0.2–0.5 g), X_4_ (catalyst loading: 5%–20%) and X_5_ (molar ratio: 3–12)] on the responses, namely the hydrogen yield, Y_1_, and the conversion of glycerol into gaseous products, Y_2_, from the steam reforming of glycerol to produce hydrogen. The experiments that were designed by CCD are shown in Table 2. Based on CCD, thirty-two sets of experiments were selected and replicated twice. These sets of experiments were not performed continuously. However, a complete experiment was carried out for each row of Table 2.

### 3.2. Statistical Analysis

The statistical package, Minitab software (version 14.12.0), was used to design the experiments and to analyze the results that were obtained from these experiments. The regression coefficients and the statistical significance of the model terms were determined by employing response surface analysis. The response surface was also applied to fit the obtained experimental data to the suggested regression model to determine the effective conditions for all response variables considered. Equation (4) below shows a second-order polynomial model, that represents the relationship between the independent and the dependent variables of the reforming process [26].

$$Y=B_{\text{0}}+\sum_{\text{i=1}}^{k}B_{i}x_{i}+\sum_{\text{i=1}}^{k}B_{ii}x_{1}^{2}+\sum \sum_{\text{i<}j}B_{ij}x_{i}x_{j}$$ (4)

where *Y* is the predicted response variable, *B*_0_ is a constant, *k* is the number of independent variables, *x* is the input variable and *B_i_*, *B_ii_*, and *B_ij_* are linear, quadratic and interaction coefficients, respectively. For five independent variables, Equation (4) becomes:

$$\begin{array}{l} Y=B_{0}+B_{1}x_{1}+B_{2}x_{2}+B_{3}x_{3}+B_{4}x_{4}+B_{5}x_{5}+B_{11}x_{1}^{2}+B_{22}x_{2}^{2}+B_{33}x_{3}^{2}\\ \;+B_{44}x_{4}^{2}+B_{55}x_{5}^{2}+B_{12}x_{1}x_{2}+B_{13}x_{1}x_{4}+B_{14}x_{1}x_{4}+B_{15}x_{1}x_{5}\\ \;+B_{23}x_{2}x_{3}+B_{24}x_{2}x_{4}+B_{25}x_{2}x_{5}+B_{34}x_{3}x_{4}+B_{35}x_{3}x_{5}+B_{45}x_{4}x_{5} \end{array}$$ (5)

The coefficients were regressed using ANOVA (*p* < 0.05), which was used as a tool to check the significance of each of the coefficients, and a reduced model was obtained by eliminating the non-significant coefficients from the first model. The R^2^ value was used to determine the fitting quality of the obtained results to the proposed model. The effects of the independent variables on the response variables were explained by three-dimensional surface plots.

### 3.3. Model Validation

To validate the models for the hydrogen yield and the conversion of glycerol into gaseous products, a basic statistical analysis using a 2-sample *t*-test was performed with Minitab. In this test, a sample in different columns, columns of experimental and predicted values given in the Minitab worksheet, was chosen to compare the fitted values of the hydrogen yield and the glycerol conversion to gases with the experimental values and to determine the accuracy of the suggested models in predicting the responses.

## 4. Results and Discussion

### 4.1. Selection of Input Variables Levels

In this study, five independent variables that could affect the reaction performance were chosen, including the temperature, the flow rate, the catalyst weight and loading and the glycerol-water molar ratio, to investigate the effects of the reaction conditions on the reforming reaction. As indicated in Table 1, the lower, upper and middle values of the mentioned independent variables were selected based on values that were obtained from the literature [30–32] and from preliminary experiments (data not shown). For the reforming temperature, the reaction performance was investigated at a wide range of temperatures from 350 to 900 °C, as reported elsewhere [14]. A thermodynamic study revealed that the high temperature > 600 °C was more favorable for hydrogen production [33,34]. However, operating at elevated temperatures would increase the energy consumption and reduce the process efficiency. In this study, the temperature variable was optimized at 400 to 600 °C to lower the energy consumption for increasing the efficiency of the reforming reaction. The results of the response variables that were obtained from the conducted experiments are given in Table 3. From these data, the best set of reaction conditions was estimated for hydrogen production (Y_1_) and the conversion of glycerol into gaseous (Y_2_) in the steam reforming of glycerol for hydrogen production.

### 4.2. Statistical Analysis

Thirty-two sets of experiments with two replicates were conducted to explore the effects of the reaction temperature, the feed flow rate, the catalyst weight, the catalyst loading and the glycerol-water molar ratio on the production of hydrogen in the glycerol steam reforming process. Prior to the reaction taking place, it should be indicated that the catalyst in all experiments was exposed to the same thermal pretreatment and reduction steps. The mean values of the obtained results, as well as the standard deviations are presented in Table 3. These results were then analyzed by analysis of variance (ANOVA) to evaluate the “goodness of fit” to the predicted models. The analysis equations generated by the first ANOVA test were reduced by removing the terms that were not statistically significant (*p*-value > 0.05). The *p*-value of a variable, when less than 0.05, indicates that the variable in the model is significant. The results that were predicted by the models are also tabulated in Table 3. Table 4 shows the regression coefficients and other statistical parameters for the reduced and non-reduced models.

For the reduced models, data obtained in this table confirmed that the reduced full quadratic models were the most significant with *p*-values < 0.05. In addition to the *p*-value, the determination coefficient R^2^ is also a good tool for judging the significance of the mathematical model by determining the goodness of fit between the model and the experimental data. A high value of R^2^ close to one indicates a more adequate representation of the data by the model. As shown in Table 4, the R^2^ and adjusted R^2^ values indicate that the predictive models satisfactorily fit the experimental data for both the responses for the hydrogen yield, Y_1_, and the glycerol conversion to gaseous products, Y_2_, and the mathematical models obtained are satisfactory.

By applying center composite design (CCD), regression equations that represent the empirical relationship between the process variables and their responses were generated. For hydrogen yield, a high R^2^ value of 0.895 suggests that the proposed model can explain most of the variations that were observed. The linear terms of the reaction temperature, X_1_, flow rate, X_2_, catalyst loading, X_4_, and molar ratio, X_5_, as well as the quadratic terms of catalyst loading, X_4_, and the molar ratio, X_5_, showed significant effects on the production of hydrogen, with *p-*values of less than 0.05. Furthermore, the interaction terms of the temperature and flow rate, X_1_X_2_, temperature and catalyst loading, X_1_X_4_, temperature and molar ratio, X_1_X_5_, and flow rate and molar ratio, X_2_X_5_ also significantly affected the hydrogen production, as indicated by ANOVA. The predicted second-order polynomial model obtained for hydrogen production (Y_1_) is expressed according to the following equation:

$$\begin{array}{l} Y_{1}=-109.5+0.142X_{\text{1}}+808.05X_{\text{2}}-1.48X_{\text{3}}+3.04X_{\text{4}}+8.8X_{\text{5}}-0.144X_{\text{4}}^{\text{2}}\\ \;-0.547X_{\text{5}}^{\text{2}}-1.43X_{\text{1}}X_{\text{2}}+0.003X_{\text{1}}X_{\text{4}}+0.005X_{\text{1}}X_{\text{5}}-19.16X_{\text{2}}X_{\text{5}} \end{array}$$ (6)

For the glycerol conversion to gases (Y_2_), the small *p-*value of the model (*p* < 0.05) and the high R^2^ value of 0.918, as shown in Table 4, indicate that the regression model that was obtained correlated with the independent variables and the conversion of glycerol into gaseous products well. The linear terms of the independent variables, X_1_, X_4_ and X_5_, the quadratic term of X_1_ and the interaction terms of X_1_X_2_, X_1_X_4_, X_1_X_5_, X_2_X_3_, X_2_X_4_ and X_2_X_5_ showed significant effects on the conversion of glycerol into gaseous products. Among these variables, the linear and the quadratic effects of temperature were the most significant variables. The regression model obtained for glycerol conversion to gases as a function of operating variables is given below:

$$\begin{array}{l} Y_{\text{2}}=-685+2.67X_{\text{1}}+568.61X_{\text{2}}-31.13X_{\text{3}}-2.54X_{\text{4}}+5.57X_{\text{5}}-0.002X_{\text{1}}^{\text{2}}\\ \;-1.36X_{\text{1}}X_{\text{2}}+0.005X_{\text{1}}X_{\text{5}}+625X_{\text{2}}X_{\text{3}}+15.5X_{\text{2}}X_{\text{4}}\\ \;-24.72X_{\text{2}}X_{\text{5}} \end{array}$$ (7)

### 4.3. Effects of Operating Conditions on Reaction Responses

#### 4.3.1. Hydrogen Yield (Y_1_)

**Figure 1.**
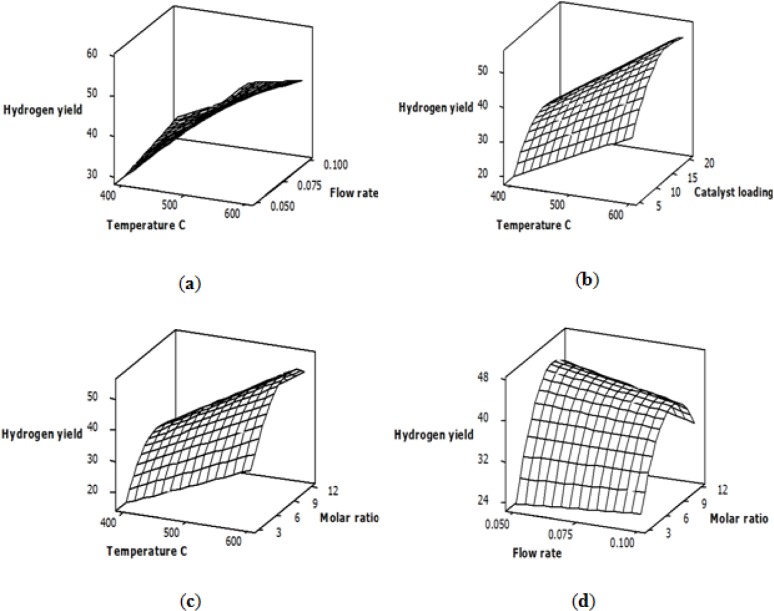
The interaction effects of process variables on hydrogen yield: (**a**) temperature and flow rate; (**b**) temperature and catalyst loading; (**c**) temperature and molar ratio; (**d**) flow rate and molar ratio.

For the hydrogen yield response, the ANOVA results shown in Table 4 indicate that the reaction temperature, the feed flow rate and the glycerol-water molar ratio have the most significant effects among the studied variables. The relationship between the variables and the hydrogen yield is explained by the response surface plots shown in Figure 1. The general trends of these plots indicate that the reciprocal interaction between the temperature and flow rate, the temperature and catalyst loading, the temperature and molar ratio and the flow rate and molar ratio have a significant effect on the hydrogen yield. The feature of using response surface plots as a function of two variables at a time is that it can describe the effects of both the main and the interaction factors.

Figure 1a–c shows the effect of varying the temperature and flow rate, the temperature and catalyst loading and the temperature and molar ratio on the hydrogen yield, while other variables are kept fixed at the central level. Plots a, b and c indicate that the hydrogen yield was increased as the temperature increased. This response is logical, because the steam reforming reaction is endothermic and favors high temperatures [35]. Plot (a) shows that at low temperatures, there was an increase in hydrogen yield when the feed flow rate was increased from 0.05 to 0.1 mL/min, while a slight decrease in hydrogen yield was observed at high temperatures, which could be attributed to an increased pressure drop inside the reactor with an increased flow rate at high temperatures. On the other hand, Plot (b) indicates that an increased catalyst loading caused an increase in the hydrogen yield. The enhanced hydrogen yield is a result of an increased number of active Ni sites dispersed on the alumina support. Plots (c) and (d) indicate that the molar ratio has a considerable effect on hydrogen yield and that its optimal value was approximately the central level of the studied values [30].

### 4.3.2. Glycerol Conversion into Gaseous Products (Y_2_)

**Figure 2.**
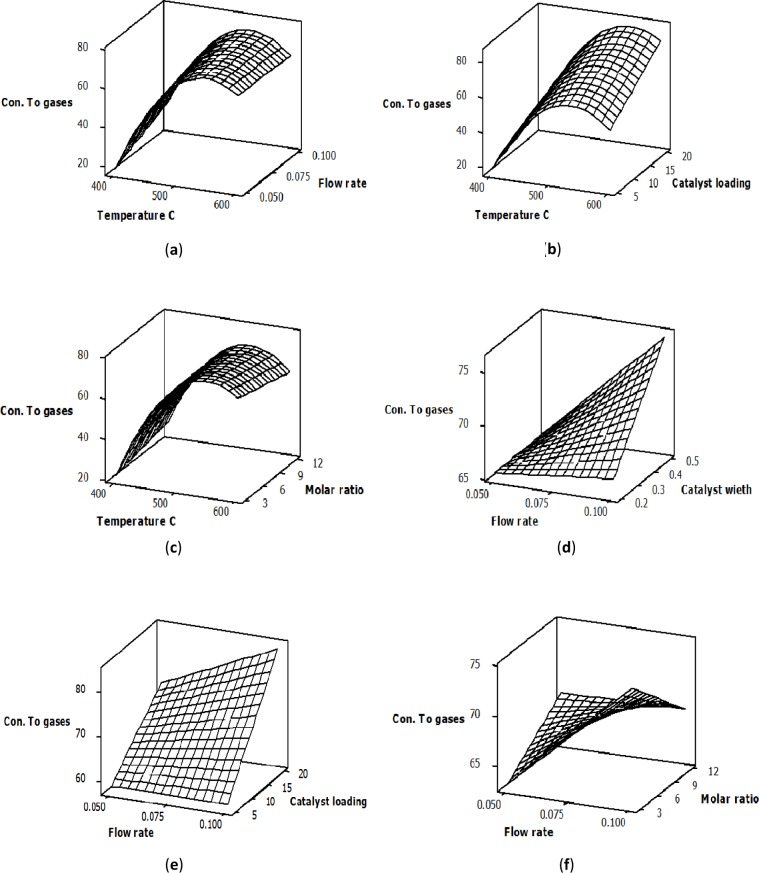
The interaction effects of process variables on glycerol conversion to gases: (**a**) temperature and flow rate; (**b**) temperature and catalyst loading; (**c**) temperature and molar ratio; (**d**) flow rate and catalyst weight; (**e**) flow rate and catalyst loading; (**f**) flow rate and molar ratio.

The response surface plots to describe the effects of both the main and the significant interaction variables on the conversion of glycerol into gaseous products over the Ni/Al_2_O_3_ catalyst is shown in Figure 2. For these figures, the orientation of the surface plots indicates that the glycerol conversion to gases was significantly affected by the mutual interaction between the variables. Plots (a), (b) and (c) show the interaction effects of the temperature and flow rate, the temperature and catalyst loading and the temperature and molar ratio, respectively. The reaction temperature had the greatest effect on the glycerol conversion to gases, which significantly increased when the reaction temperature increased from 400 to 600 °C. The improvement in glycerol conversion to gases is a result of the endothermic steam reforming reaction. At a constant temperature, there was no significant change in the conversion of glycerol into gaseous products with a change in flow rate or molar ratio, as shown in Plots (a) and (c).

However, a considerable increase in glycerol conversion to gases with an increased catalyst loading at constant temperature was observed, as shown in Plot (b). This increase in glycerol conversion to gases could be attributed to the increase in the catalyst surface area, which provides more contact area between the glycerol molecules and the active sites of the Ni catalyst. The response surface plots in (d) and (e) explain the interaction between the glycerol conversion to gases and both the flow rate and the catalyst weight, as well as the flow rate and the catalyst loading, respectively. Both plots indicate that the feed flow rate did not have any significant effects on the glycerol conversion to gases at low catalyst weight and low catalyst loading. On the other hand, at higher levels of catalyst weight and catalyst loading, the conversion of glycerol into gaseous products increased as the flow rate increased. This enhancement in the glycerol conversion to gases with an increased flow rate could be explained by the increase in the surface area of the active sites on the Ni catalyst when the catalyst weight and catalyst loading increased from 0.2 to 0.5 g and from 5% to 20% correspondingly. Plot (f) in Figure 2 shows the interaction effect of the feed flow rate and the glycerol-water molar ratio on glycerol conversion to gases. The orientation of the principal axes of the surface plot indicates that the interaction between the feed flow rate and the glycerol-water molar ratio significantly affected glycerol conversion to gases and that the optimum values of both variables existed within the area that was experimentally explored.

### 4.4. Response Optimization

In this work, the optimal temperature, flow rate, catalyst weight, catalyst loading and glycerol-water molar ratio were evaluated as a combination of both responses to obtain a high reaction performance for the reforming of glycerol over a Ni/Al_2_O_3_ catalyst for hydrogen production. The best values of these variables that led to desirable response goals were determined by conducting multiple numerical and graphical optimizations. The overall effective conditions, based on the response surface methodology for hydrogen production, were at a temperature of approximately 600 °C, a feed flow rate of 0.05 mL/min, a catalyst weight of 0.2 g, a catalyst loading of 20% and a glycerol-water molar ratio of approximately 12. By applying the obtained optimal conditions, the predicted response values of hydrogen yield and the conversion of glycerol to gaseous products were 57.6% and 75%, respectively.

### 4.5. Model Validation

A hypothesis test was performed to compute a confidence interval of the difference between the experimental and the fitted data for both the hydrogen yield and the glycerol conversion to gases responses using a two-sample *t*-test offered by Minitab. In this test, the effectiveness of the proposed models of Equations (6) and (7) was explored by determining whether the difference between the experimental and predicted results was zero. Moreover, the *p*-value is a good tool that can be used to check the validity of the models. Table 5 displays the sample size, means, standard deviations, standard errors, 95% confidence intervals for the difference and *p*-values for both responses. As shown in the table, the 95% confidence interval was (−5.11, 5.11) and (−7.67, 7.67) for H_2_ yield and glycerol conversion to gases, respectively. As zero lies between these intervals, the 95% CI indicates that there is no difference between the experimental and the fitted results, therefore showing that the models are valid for representing the responses. Furthermore, the *p*-value for both responses was much greater than 0.05, indicating that there was no difference and that the suggested models could be used to explain the variation of the studied variables.

## 5. Conclusions

In this work, the steam reforming of glycerol for hydrogen production was performed over a Ni/Al_2_O_3_ catalyst. To maximize hydrogen yield and glycerol conversion to gases, a statistical method was effectively employed to analyze the variation effects of the process variables. Five process parameters, including the reaction temperature, the feed flow rate, the catalyst weight, the catalyst loading and the glycerol-water molar ratio, were investigated using RSM. The ANOVA results demonstrated that the reaction temperature, X_1_, the feed flow rate, X_2_, the catalyst loading, X_3_, and the glycerol-water molar ratio had significant effects on the responses. For the catalyst weight, no significant effect was observed, which could be attributed to: (1) the small size of the reactor or (2) the low flow rate that was used. Furthermore, a second-order regression equation was derived for the hydrogen yield and the glycerol conversion to gases responses. The response optimizer revealed that the optimum values of the studied variables were a temperature of approximately 600 °C, a feed flow rate of 0.05 mL/min, a catalyst weight of 0.2 g, a catalyst loading of 20% and a glycerol-water molar ratio of approximately 12. At these conditions, the maximum hydrogen yield and glycerol conversion to gases were predicted to be 57.6% and 75%, respectively. A two-sample *t*-test was performed to check the validity of both models. The results obtained by the test indicated that the models were of high accuracy, with the capability of predicting the responses within the range of investigated variables. Based on the obtained results, it can be concluded that RSM is a good tool that can evaluate the effects of separate and interacting variables on the process response while requiring less experiments.

## Figures and Tables

**Table 1.** Coded value of the independent variables.

Independent variables	Symbol	Coded values		
		**−**1	0	1
Temperature (°C)	X_1_	400	500	600
Flow rate (mL/min)	X_2_	0.05	0.075	0.1
Catalyst weight (g)	X_3_	0.2	0.35	0.5
Catalyst loading (%)	X_4_	5	12.5	20
G:W molar ratio	X_5_	3	7.5	12

**Table 2.** The central composite design for proposed work.

Run order	Pt type	Temperature (°C)	Flow rate	Catalyst weight	Catalyst loading	Molar ratio
1^(C)^	0	500	0.075	0.35	12.5	7.5
2	1	400	0.05	0.5	20	12
3	1	400	0.1	0.5	20	3
4^(C)^	0	500	0.075	0.35	12.5	7.5
5	1	400	0.1	0.2	5	3
6	1	400	0.05	0.5	5	3
7	1	600	0.05	0.5	20	3
8	1	600	0.1	0.5	20	12
9	−1	500	0.075	0.35	12.5	3
10	−1	500	0.075	0.35	20	7.5
11	1	600	0.1	0.2	5	12
12	−1	500	0.075	0.35	12.5	12
13	−1	600	0.075	0.35	12.5	7.5
14	−1	500	0.05	0.35	12.5	7.5
15^(C)^	0	500	0.075	0.35	12.5	7.5
16	1	600	0.05	0.5	5	12
17	1	400	0.05	0.2	20	3
18	1	400	0.05	0.2	5	12
19	1	600	0.05	0.2	5	3
20	−1	500	0.1	0.35	12.5	7.5
21	1	600	0.1	0.5	5	3
22^(C)^	0	500	0.075	0.35	12.5	7.5
23^(C)^	0	500	0.075	0.35	12.5	7.5
24^(C)^	0	500	0.075	0.35	12.5	7.5
25	1	400	0.1	0.2	20	12
26	−1	500	0.075	0.2	12.5	7.5
27	1	600	0.1	0.2	20	3
28	−1	400	0.075	0.35	12.5	7.5
29	1	600	0.05	0.2	20	12
30	−1	500	0.075	0.35	5	7.5
31	−1	500	0.075	0.5	12.5	7.5
32	1	400	0.1	0.5	5	12

(C) center point.

**Table 3.** The results data for the independent variables obtained from the designed experiments.

Run order	Pt type	H_2_ yield (Y_1_)	Predicted value for (Y_1_)	Glycerol con. to gases (Y_2_)	Predicted value for (Y_2_)
1^(C)^	0	45 ± 0.71	42.2	76 ± 0.49	68.4
2	1	23 ± 0.08	21.1	30 ± 0.15	27.3
3	1	13 ± 0.46	16.3	45 ± 2.8	44.1
4^(C)^	0	45 ± 0.56	42.2	76 ± 0.85	68.4
5	1	7 ± 0.78	8.7	12 ± 0.71	17.3
6	1	6 ± 0.18	−0.49	10 ± 0.14	9.7
7	1	30 ± 2.1	35.2	76 ± 0.26	76.8
8	1	35 ± 0.37	42.9	75 ± 0.96	78.8
9	−1	27 ± 0.68	24.4	72 ± 1.2	68.7
10	−1	47 ± 1.4	40.2	74 ± 0.21	78.2
11	1	27 ± 0.5	27.1	37 ± 0.27	35.9
12	−1	38 ± 0.48	37.8	60 ± 0.52	68.1
13	−1	55 ± 0.7	52.5	78 ± 2.12	64.4
14	−1	29 ± 0.5	43.5	53 ± 0.39	65.5
15^(C)^	0	45 ± 1.2	42.2	75 ± 1.3	68.4
16	1	42 ± 1.4	40.9	55 ± 0.37	53.1
17	1	8 ± 0.35	8.0	14 ± 0.77	15.5
18	1	12 ± 0.71	13.4	22 ± 0.5	21.4
19	1	20 ± 1.00	19.5	53 ± 0.33	54.9
20	−1	48 ± 0.8	40.8	70 ± 2.1	71.2
21	1	12 ± 1.4	13.4	52 ± 0.4	58.3
22^(C)^	0	44 ± 0.24	42.2	76 ± 1.4	68.4
23^(C)^	0	44 ± 0.75	42.2	76 ± 0.83	68.4
24^(C)^	0	44 ± 0.56	42.2	75 ± 0.85	68.4
25	1	23 ± 0.09	21.7	29 ± 0.63	35.3
26	−1	38 ± 0.55	42.4	71 ± 1.4	66.0
27	1	31 ± 0.62	30.1	82 ± 0.33	82.4
28	−1	20 ± 0.22	31.9	25 ± 0.71	24.7
29	1	61 ± 0.62	57.6	72 ± 0.1	75
30	−1	23 ± 0.3	28.0	47 ± 2.1	58.5
31	−1	47 ± 0.54	42.0	56 ± 0.1	70.7
32	1	15 ± 0.06	13.1	33 ± 0.24	27.3

(C) center point.

**Table 4.** Full model and reduced model coefficient parameters for both responses.

Regression coefficient	Symbol	Full model				Reduced model			

		Parameter estimate		*p*-value		Parameter estimate		*p*-value	

		H_2_ yield (Y_1_)	Glycerol con. to gases (Y_2_)	H_2_ yield (Y_1_)	Glycerol con. to gases (Y_2_)	H_2_ yield (Y_1_)	Glycerol con. to gases (Y_2_)	H_2_ yield (Y_1_)	Glycerol con. to gases (Y_2_)
Linear	X_1_	0.56	1.77	0.021	0	0.142	2.672	0	0
	X_2_	1578.73	1674.43	0.011	0.025	808.05	568.611	0	0.069
	X_3_	49.98	115.2	0.544	0.247	−1.481	−31.134	0.805	0.244
	X_4_	2.56	0	0.055	0.999	3.04	−2.539	0.008	0.014
	X_5_	7.13	6.12	0.002	0.024	8.8	5.571	0	0.001

Quadratic	X^2^_1_	0	0	0.105	0	–	−0.002	–	0
	X^2^_2_	−4532.2	−7372.11	0.228	0.105	–	–	–	–
	X^2^_3_	62.99	−138.11	0.544	0.27	–	–	–	–
	X^2^_4_	−0.1	−0.09	0.016	0.088	0.144	–	0	–
	X^2^_5_	−0.44	−0.01	0	0.969	−0.547	–	0	–

Interaction	X_1_X_2_	−1.44	−1.36	0	0.003	−1.43	−1.362	0	0.009
	X_1_X_3_	−0.11	−0.12	0.075	0.11	–	–	–	–
	X_1_X_4_	0	0.01	0.031	0.001	0.003	0.005	0.037	0.002
	X_1_X_5_	0	−0.01	0.026	0.003	0.005	−0.008	0.032	0.009
	X_2_X_3_	−241.67	625	0.324	0.037	–	625	–	0.069
	X_2_X_4_	−0.5	15.5	0.918	0.011	–	15.5	–	0.025
	X_2_X_5_	−19.17	−24.72	0.022	0.014	−19.16	−24.722	0.027	0.032
	X_3_X_4_	−1.58	0.25	0.056	0.798	–	–	–	–
	X_3_X_5_	−0.32	0.88	0.811	0.589	–	–	–	–
	X_4_X_5_	0.01	−0.06	0.707	0.062	–	–	–	–

R^2^		92.0%	94.8%	–		89.5%	91.8%	–	
R^2^ adjusted		88.3%	92.4%			87.2%	89.9%		

**Table 5.** Two-sample *t*-test for both models.

Response	N	Mean	SD	SE mean	95% CI	*p*-value
Experimental H_2_ yield	64	31.5	15	1.9	(−5.11, 5.11)	1.00
Fitted H_2_ yield	64	31.5	14.2	1.8		
Experimental glycerol con.	64	55	22.4	2.8	(−7.67, 7.67)	1.00
Fitted glycerol con.	64	55	21.5	2.7		
